# Determinants of Sailors' Protective Behaviors in Fishing Spots against the Risks of Sunlight and Skin Cancer: A Qualitative Study in Iran

**DOI:** 10.1155/2021/9954946

**Published:** 2021-07-17

**Authors:** Ali Asadian, Mojtaba Fattahi Ardakani, Ahmad Sotoudeh, Moradali Zareipour, Ehsan Movahed

**Affiliations:** ^1^Social Determinants in Health Promotion Research Center, Hormozgan Health Institute, Hormozgan University of Medical Sciences, Hormozgan, Iran; ^2^Diabetes Research Center, Shahid Sadoughi University of Medical Sciences, Yazd, Iran; ^3^Department of Public Health, Bushehr University of Medical Sciences, Bushehr, Iran; ^4^Health System Research Unit, Urmia Health Center, Urmia University of Medical Sciences, Urmia, Iran; ^5^Health Education and Health Promotion, School of Public Health, Jiroft University of Medical Sciences, Jiroft, Iran

## Abstract

**Introduction:**

The growing rate of nonmelanoma skin cancer (NMSC) and melanoma has been a great challenge for global health system. The present research aims to determine sailors' protective behaviors against the risks of sunlight and skin cancer in Iran.

**Materials and Methods:**

The present research was qualitative in type, and its data were collected from August to December 2019. To this aim, 23 participants were recruited with whom semistructured interviews were held. The data collection continued until data saturation, and the interviews were coded in MAXQDA 10. Conventional content analysis was used to analyze the qualitative data.

**Results:**

Analysis of sailors' perceptions and experiences revealed 7 categories: protective behaviors, hard personal and familial conditions, social interactions, poor social support, feeling of satisfaction, self-care, and fear.

**Conclusion:**

Sailors are prone to skin cancer due to their specific work conditions. Perceptions and determinants of skin cancer and protective behaviors against sunlight were identified among sailors. Promotion of protective behaviors and beliefs that impeded preventive behaviors are among issues that require special attention.

## 1. Introduction

A key general health issue in the world is the increasing rate of NMSC and melanoma (1). NMSC is the most prevalent type of skin cancer around the globe [1]. As the body of related research shows, annually about 2-3 million new cases of NMSC are reported worldwide [2]. The adverse effects of the ultraviolet radiation on skin and the chances of skin cancer are known for decades. Long-term exposure is a main factor involved in sunburn and skin cancer [3]. Moreover, UVR has been categorized as a cancerous material for humans and a factor involved in melanoma and NMSC [4]. Sailors are always exposed to a high level of UVR. The related literature has provided epidemiological evidence for the high risk of external workers and the chances of NMSC compared to internal workers [5].

In addition, as the related body of research shows, constant exposure to UVR is strongly correlated with the risk of NMSC [6, 7]. Unfortunately, for external workers, it is very common to be exposed to sunlight at the midday when the UVR is intense. The body of national research has indicated an increasing rate of skin cancer in Iran especially in sailors for whom the incidence rate of skin cancer is high due to constant exposure to UVR and a higher intensity of sunshine [8, 9]. Sailors, due to their work conditions, spend long hours on sea and are, thus, largely exposed to the sunlight, which increases the chances of affliction with skin cancer in the long run [9, 10]. Finally, in 2015, skin cancer was recognized as an occupational disease globally [11].

Developing goal-oriented and effective interventions to promote protective behaviors against sunlight where workers work outdoors is of a great importance. While developing health interventions, to initiate a change in behavior, attitude, and beliefs of the target groups, knowing perceptible barriers is significant (e.g., time pressure for the adoption of protective behaviors) [12]. Besides primary prevention, it is essential to use secondary preventive measures to reduce the load of the disease through a timely diagnosis and adequate treatment of the disease. Therefore, to evaluate and lower the burden of the disease, it is essential to evaluate the exposure of external workers to UVR and the required preventive measures [13]. Therefore, it is necessary to know sailors' perceptions and experiences adequately as these might significantly affect health-related behavior change. That is why an awareness of perceived threat and motivation for protection should be considered as a key factor [14].

The extent to which skin cancer preventive behaviors are adopted does not suffice for health. It seems that different factors affect this healthy behavior but have remained largely unknown. To reveal the different overt and covert aspects of skin cancer preventive behaviors, it is necessary to explore sailors' perceptions and experiences first so as to attain a real understanding of their experience. This will be used as a basis to develop an appropriate educational program to meet the needs. A body of research dealt with external workers' perceptions of protective behaviors against UVR. Among them was some research conducted by Zink et al. on farmers. It revealed the perceived personal barriers effective in taking sun protective measures and personal experiences and special living conditions on farms as the key concepts involved in farmers' perceptions of keratinocyte cancer (KC) primary preventive behaviors [15]. As Rocholl et al. investigation revealed, external workers did not protect their head and neck thoroughly. They perceived wearing long sleeves and long pants hard and did not tend to wear a sunscreen [16].

To our knowledge, there has been less research into external workers' experiences and protective behaviors and no research has been done on sailors. Besides, the determinants of external workers' protection against UVR especially sailors' perceptions can largely contribute to the design of interventions to promote protective behaviors. The present research aimed to explore sailors' experiences and perceptions so as to learn about the barriers and facilitators of protective behaviors to illuminate prospective interventional programs.

## 2. Materials and Methods

### 2.1. Research Design and Participants

The present qualitative research employed a guided content analysis and was conducted from August to December 2019. The research population consisted of all sailors working in fishing spots in Bandar Bushehr, Iran. The inclusion criteria were at least one year of sea work experience, residence in Bandar Bushehr, and having no skin cancer oneself or among close relatives. Exclusion criteria were inability to communicate, miscommunication of experiences, and lacking cooperation in the interview. The sampling method was purposive in type and based on the inclusion criteria. The data collection continued until data saturation.

### 2.2. Data Collection

Having obtained the required permissions from the ethics committee of Yazd University of Medical Sciences and the Bureau of Harbors and Shipping in Bandar Bushehr, the sampling was done in three spots. The purpose of research was explained to the participants, and they were all ensured of the confidentiality of the information they provided. Semistructured interviews were used to collect the data. The main questions asked were the following: “please share the experience of one working day while protecting against sunlight” or “please tell us about your experience of preventing skin cancer.” Then, the interview continued according to the interviewer's previous response to delve into more details with probes such as the following: “what do you mean?” or “can you explain more?” The general questions in the interview addressed demographic information, and the interview guide was based on the protection motivation theory. Interviews were held with 23 participants and continued until data saturation. They were held face to face at the seashore or in the fishing spots. The interviews took between 30 and 55 minutes depending on the conditions. They were audio-recorded and then transcribed. The content was later on typed word by word.

### 2.3. Data Analysis

Data analysis was done following the method proposed by Graneheim and Lundman [17]. In this method, the interviews were read more than once until an overall impression was gained from the content. Then, the content of participants' experience of preventing skin cancer was analyzed and the units of analysis were determined. Then, the text was divided into meaning units. After abstracting the meaning units, the coding was done. The codes were divided into a number of categories and subcategories in terms of differences and similarities. The coding was done to extract the main themes from sailors' experiences and determine the scale constructs. To manage the qualitative data, MAXQDA10 was used. Data analysis began with the first interview and continued so forth.

### 2.4. Rigor

To test the rigor of data, the criteria suggested by Guba and Lincoln were used [18]. For data accuracy and validity, enough time was spent on data collection and analysis. The participants were contacted effectively to acquire the information needed. A variety of data were used to gain more in-depth information. Constant comparison of data and revision of codes were done by a team of experts to ensure data credibility. To increase dependability, the process of qualitative analysis and content analysis was followed and mapped in detail. Besides, to increase authentication, at the end of each interview, a summary of what the interview obtained from the whole interview was restated for the participants to control the accuracy of content. During the data analysis and categorization, the extracted categories and codes were shown to some participants to check the accuracy of tags and codes. To increase the transferability of data, it was attempted to fully describe the participants' culture and background as well as data collection and analysis procedure.

### 2.5. Ethical Considerations

The present research was approved by the ethics committee of Shahid Sadoughi University of Medical Sciences in Yazd (IR.SSU.SPH.REC.1397.085). The written form of informed consent was obtained from each participant. They were all allowed to leave the study voluntarily whenever they wanted. They were ensured of the confidentiality of the information they provided. The interviewees' names were not written on records and texts.

## 3. Results

The majority of participants aged between 20 and 50 years. The mean age was 38 ± 1.5 years.  Table 1 summarizes the participants' demographic information.

The analysis of Iranian sailors' perceptions and experiences led to the extraction of 7 categories and 15 subcategories of skin cancer (Table 2).

## 4. Protective Behaviors

Reduced exposure to UVR (in the sunlight) and showing protective behaviors are among the primary preventive measures of skin cancer. To this aim, appropriate attempts and activities can act as the first step of self-protection. This category is made up of two subcategories: change of work time and use of protectives.

About half of the participants stated that changing the work time is an appropriate way of reducing exposure to sunlight or sunburns. Here is an extract from a participant's account: “Most of the time, I tried to go fishing at night. Many nights especially in summer, we went anchoring, which was done successfully. It was truly effective” (a 47-year-old sailor).

Another participant commented, “When the sun is shining intensely especially in summer, we do not go to the sea. It is very hot always and in the sea, it is the hottest” (a 39-year-old sailor).

A few participants said that intense sunlight and extremely hot weather made sailors protect themselves more with suitable clothes and hats. Doing so, they lowered the chances of sunburn:

“I myself, most of the time especially when it is too hot, wear a hat. It is so hot that wearing hats is unavoidable. So, I wear hats with shades all around” (a 39-year-old sailor).

“Since I go to the sea, I wear suitable clothes most of the time. I always wear long sleeves which has proved useful. Here, the sunlight is burning hot” (a 41-year-old sailor).

## 5. Hard Personal and Familial Conditions

Occupational and familial status is based on social and economic support and affects spirits tremendously. Sailors, when faced with a lower income and a risk of losing job and family relations, develop negative feelings. This category consists of three subcategories: loss of peace, loss of work and family status, and loss of health.

Many sailors said that affliction with skin cancer gave them the feelings of misgiving and disappointment. Also, when they see cancerous patients, they feel more pain and depression.

“Cancer is an intolerable disease. One gets desperate and not feeling like doing anything” (a 27-year-old sailor).

“Cancer is truly dreadful as it makes people sad and confused. When you hear about it, your whole body trembles. For me, it is intolerable. Too hard” (a 48-year-old sailor).

“Cancer disables people. It weakens and handicaps them. You cannot handle your personal stuff. Someone should always help you” (a 35-year-old sailor).

A lot of sailors were unable to earn a living. They showed concerns about being as active and capable of before. They were unable to earn a living as they used to. Here is a relevant comment:

“I go to the sea three to four times a week. It depends on the weather. If it is good, I go there more often and earn a living this way. It's hard to believe. When you get down with cancer, you cannot be the person you used to be. You will not be able to do anything” (a 42-year-old sailor).

Most sailors correlated the outcomes of skin cancer and unemployment with inability to earn a living. They showed concerns about their family occupation and economy.

“My family depend on me. This is how I earn a living. If we are sick, we will be a burden on our family. Now, in hard days, if there is no income, how can we live? If the breadwinner is unable to work anymore, he will be too ashamed to face the family” (a 37-year-old sailor).

About half of the participants pinpointed the ineffectiveness of measures and the deadly nature of the disease. Here is an instance:

“Skin cancer is really dangerous. You should spend all your money on visits to doctors and at the end you gain nothing. There is no cure. Whoever is afflicted will die soon afterward” (a 68-year-old sailor).

“Cancer is very serious and dangerous. Its name frightens everyone. Whoever gets afflicted will die for sure. There is no cure” (a 45-year-old sailor).

## 6. Social Interactions

Encouragement, on the one hand, promotes a behavior and acts as an external factor and, on the other hand, has a certain purpose and outcome and when repeated it turns into an internal factor. According to the participants' experiences, the social interactions category was found with 2 subcategories: demotivation and discouragement and rejection.

As an instance, a number of participants commented the following:

“No; nobody talked about this to us, neither our doctor nor the health staff. If it was good, they for sure told us. When nobody told us about it, how could we know?” (a 39-year-old sailor).

“This weather we have here exhausts us. This exhaustion demotivates us to show these behaviors. I, in person, am not interested in doing such stuff. Many things do not fit us at all, like wearing sunglasses in the sea” (a 28-year-old sailor).

“I remember when I was engaged, I used to wear a sunscreen. When a colleague saw me, he made fun of me and said these things were girlish and had nothing to do with our job. I remember he laughed at me; still remember it” (a 32-year-old sailor).

“About two years ago when it was too hot, I remember wearing a hat and scarf and fully covered myself. When I reached the harbor and got into a boat, my friend picked on me and asked why I covered myself like that and that I could not work and I could easily get distracted. He said to colleagues in the boat nearby that I was feeling cold and they all laughed at me” (a 32-year-old sailor).

## 7. Poor Social Support

A lack of social support on the one hand and negative attitude to preventive behaviors on the other can hinder or stop protective behaviors. Thus, attempts should be made to correct negative attitudes to favor continuous protective behaviors. As the sailors' experiences showed, the social support category had two subcategories: issues with living and a negative attitude to protective behaviors.

Some sailors mentioned financial problems, responsibility of family, and earning a living among their main duties. Thus, for them, meeting the primary needs of a living and making money were priorities in life.

“Our income is low. Yet, for sure, now making a living is our priority. I cannot now think of my health. All prices are tremendously high. Nothing is fixed in the market. I should have my own job and make a living” (a 45-year-old sailor).

“I am the breadwinner and should pay for everything. I have a family to feed. The costs are high. I should find a way to pay for the prices” (a 40-year-old sailor).

“I think if people do not find it uneasy, they should wear a sunscreen. But an old friend who wore sunscreen before said that when he sweated, it burned his eyes and hurt his skin and, thus, he stopped wearing it. But, I myself have not yet experienced it” (a 37-year-old sailor).

A participant said that wearing long sleeves produced blisters on skin or made it red and that they could not tolerate hot weather in such clothes.

“I feel more convenient like this. I remember the first years I wore long sleeves several times and also a scarf. But it got worse, I used to sweat which burned the skin more. Blisters appeared on my skin and it got red. So, now I feel much better like this” (a 44-year-old sailor).

## 8. Feeling of Satisfaction

This is concerned with sailors' perception of whether protective behaviors can eliminate the risk of skin cancer or not. Therefore, the effectiveness of the suggested behaviors should be explained to the participants. Their experiences of this category were further divided into two subcategories: feeling healthy by showing protective behaviors and preventing the development of disease or lowering costs by showing protective behaviors.

Many participants stated that showing protective behaviors against the sunlight not only ends in high spirits and peace but also reduces the adverse effects of exposure to sunlight including a sunburn, blister, or redness. Here is an extract:

“When I take a good care of myself, I will keep diseases away. I feel happy and in higher spirits” (a 36-year-old sailor).

“Most often, I try to take care of my skin. I see around that colleagues get older sooner than others. One reason for that is their hard work. Another reason can be the sunlight and hot weather. Their face is full of wrinkles. The get old soon. When I see these things, I tend to be more careful” (a 27-year-old sailor).

“When I had blisters, I visited a doctor. Thanks God, it was nothing serious. The sooner you take care of yourself, the sooner you get better and the less money you spend” (a 40-year-old sailor).

“When you do what you need to do and visit a doctor, you will be sooner cured and you spend less money” (a 6-year-old sailor).

### 8.1. Self-Care

Self-care involves activities that involve an individual using his/her own skills, knowledge, and capabilities as a source to take care of his/her health. The participants' experiences have been discussed here in 2 subcategories: capability of self-care and strengthening beliefs.

“I, almost always, wear hats with shades all around. Even when the sea is stormy, I wear a hat too. I tighten the hat string. It really helps not to have my neck or head sun-burned. Sometimes we go to the sea at night” (a 33-year-old sailor).

“When it gets warmer and the mid-day approaches, especially in summer, we go to the sea less. Usually, when it is cool, the sea has a special warmth. But in hot days, I get less engaged” (a 27-year-old sailor).

Any attempt to promote knowledge and beliefs is the basis of behavior change. A few participants attempted to strengthen beliefs in the prevention of skin cancer:

“What matters is that we lack enough knowledge of this. I myself sometimes try to get information about skin cancer. It is for sure good. If we know more, we take a better care” (a 32-year-old sailor).

“Your colleague helps us with that. Most often, we need to visit a doctor to give us a test. It will be a good chance to ask the doctor what we can do not to be afflicted with cancer” (a 32-year-old sailor).

## 9. Fear

If people understand that they are at a risk of a serious health threat, a higher level of fear arises, and, thus, they get more motivated to show preventive behaviors. Sailors' experiences of this category are discussed along two subcategories: fear of affliction and continuous concerns.

A number of participants acknowledged that they experienced much fear and concerns upon seeing their colleagues' problems, work conditions, and exposure to sunlight.

“Because my neighbor was on the sea for years and got afflicted with a skin cancer, I am really afraid of the disease. I have heard there is no cure to it which frightens me more” (a 42-year-old sailor).

“How come we know for sure that we never get the disease? We work in the sunlight which is really frightening. Now that I am talking about it, I am scared. It will really busy us” (a 40-year-old sailor).

A peaceful environment, informed behavior, and use of correct information comprise the main factor in preventing skin cancer. A relevant comment is as follows:

“I am really worried about my life. I am afraid of not being able to earn a living. For sure, my family will suffer. Your days and nights will be ruined. You will all be worried and everything gets disrupted” (a 45-year-old sailor).

“What worries me most is that even your friends leave you. Your family might also leave you. This is what we humans are like. You end up lonely” (a 49-year-old sailor).

Besides, a participant complained about being forgotten by others and the resultant feeling of isolation and aloofness.

“Exactly, it all begins from here. When you get afflicted with cancer, at the beginning, everything might sound alright but little by little you will be deserted” (a 65-year-old sailor).

## 10. Discussion

The present qualitative research explored sailors' experiences and perceptions of skin cancer while being largely prone to the sunlight. Overall, the analysis of qualitative data led to the extraction of 7 categories: protective behaviors, hard personal and familial conditions, social interactions, poor social support, feeling of satisfaction, self-care, and fear. About half of the participants stated that a change of work time was an appropriate way of reducing exposure to sunlight and the consequent sunburn. To confirm these findings, German workers attempted to begin their work time early in the morning in summer. Wearing a sunscreen and exchanging information about protection against sunlight is considered as a sign of intelligence and awareness among workers [19]. In their research, Rodrigues et al. reported the use of protectives at the peak time of sunlight from 11:00 a.m. to 3:00 p.m. among a few workers [20]. Avoiding sunlight between 10:00 a.m. and 4:00 p.m. to prevent sunburns should be integrated as a preventive strategy of skin cancer among sailors.

A few participants stated that intense sunshine and hot weather helped them take a good care of themselves by using protectives such as scarves and hats. This would impede the chances of sunburns. The present findings are consistent with the work of research by Rodrigues et al. The participating sailors in this research were motivated enough to protect themselves against sunlight and UVR. Yet, they did not seem to be effectively involved in taking protective measures [20]. In contrast, in their research, Zink et al. reported wearing long pants and scarves as an effective protective measure among external workers [13]. In American farmers compared to nonfarmers, use of protectives such as long sleeves showed to be more common [21]. In some other research, Mazloomy et al. reported the use of sunscreen as the most common protective measures among university students [22]. It seems that using protectives such as a scarf and a hat has turned into a convention among external workers including sailors and other target populations. Avoiding threatening factors can help prevent the skin cancer. Wearing adequately covering clothes such as light long-sleeved shirts or long pants, hats with wider edges, and sunglasses as well as a sunscreen can significantly affect the prevention and control of skin cancer.

Many sailors stated that if afflicted with the skin cancer, they feel worried and disappointed. Depression and sadness were among other feelings they claimed they would experience. A study that confirmed this finding was conducted by Mazloomy et al. among university students, who perceived skin cancer a threat [22]. Another study in American adults showed that when diagnosed with melanoma, people experience a shock, fear, and anxiety. Other problems were also reported in their family relations, disappointment, and lack of mutual understanding by others as the undesirable outcomes of skin cancer [23]. A unique code in this research concerning the loss of job and family included the loss of one's status in family, feeling humiliated in family, and unemployment. These codes show sailors' sense of belonging, interdependence, and responsibility of earning a living and managing a family. About half of the participants mentioned such issues as the effectiveness of protective measures and the fatality of the disease. In the body of research on the public, skin cancer showed to be perceived as fatal, fearsome, and incurable [20–24]. American farmers also confirmed this finding and reported skin cancer to be deadly [21]. The present findings are in contrast with Rodrigues et al., who referred to exposure to sunlight as a source of supplying vitamin D and curing diseases such as depression [20]. To reduce exposure to the sunlight and increase protective behaviors, it can be useful to emphasize the short-term effects of exposure to sunlight including dried skin, blisters, wounds, and sunburn in educational programs.

Demotivation and discouragement to show protective behaviors, miscommunication, and inadequate emphasis on such behaviors on the part of the health staff were among other issues that sailors raised. In contrast, English and Australian university students' experience was indicative of adequate motivation to show protective behaviors. The role of peers, media, family, and childhood habits was significant in motivation [15–19]. It seems that doctor's advice is a key motivating factor in showing protective behaviors. Thus, through changing an individual's beliefs, it is possible to change the emotional and affective load of the disease and expect the emergence and continuation of a healthy behavior. The majority of participants pinpointed the health staff's inadequate emphasis on protective behaviors and the lack of personal tendency to such behaviors. The role of peer pressure in accepting or rejecting behaviors is inevitable in encouraging or discouraging a behavior. An interventional strategy to promote protective behaviors is attention to sailors' peers and colleagues. Sailors' colleagues and family should be involved in the development and implementation of educational programs.

About half of the sailors had erroneous beliefs about protective behaviors and their ineffectiveness in the lower risk of skin cancer. An instance is the ineffectiveness of wearing sunscreen or long sleeves. This finding is consistent with Rodrigues et al., who drew attention to the inessentiality of sunscreen, unease of wearing sunscreen, thought of the chemical content of sunscreen, and unease of wearing long clothes [21]. On the contrary, Mazloomy et al. reported that 63% of the participants rejected the idea that the sunscreen has no effect on preventing skin cancer [25]. These contradictory findings can be due to the different target research groups and the participants' awareness. Thus, in order to raise sailors' awareness and correct their erroneous beliefs of the essentiality of protective behaviors, educational strategies need to be implemented. It is recommended to apply a combination of protective behaviors that can be effective in preventing skin cancer.

The best benefit, as perceived by the participants, has been the positive effects on health, convenience, and prevention of the development of the disease and reduction of the associated costs. This finding was consistent with the research by Baghiani Moghadam et al., whose participants revealed a high awareness of the benefits of avoiding risky behaviors associated with gastric cancer [26]. In the research by Zink et al., female farmers had a more positive perception of protection against sunlight. Besides, they were better aware of the significance and efficiency of skin cancer preventive behaviors [5]. Contrary to the present findings, Bryant et al. found that Australian adolescents did not perceive the essentiality of protective behaviors against sunlight to prevent skin cancer [27]. It seems that these divergent findings are due to the different target research groups. Adolescents often do not perceive themselves at the risk of a disease and the health issue might not be their priority.

In the present research, the majority of participants admitted that they were incapable of planning for protective behaviors and were not even capable of obtaining the sources and conditions required for the adoption of protective behaviors to improve health. This finding was consistent with Tyrel et al. who evaluated the motivation information model and behavioral skills for amphetamine consumption and medical adherence. This research showed that medical self-efficacy was very low among patients [28]. In their research, Rodrigues et al. showed that participants were truly motivated to protect themselves against sunlight and took safety messages seriously. They took a good care of themselves [20]. These findings show that self-care is primarily affected by awareness and the improvement of communicative skills to promote skin cancer preventive behaviors [29–31].

Most participants believed that due to the current work conditions and exposure to sunlight, they stood higher chances of affliction in future. This was frightening to them. This finding was consistent with the findings reported by Vogel et al. who observed that most participants experienced a shock, fear, and epilepsy when the skin cancer was diagnosed. Some physical inconveniences were also reported such as pain, numbness, and lymphedema. Yet, some others reported none and rather experienced affective problems such as anxiety, loneliness, and isolation [23]. If people believe that they are at a risk of a serious threat to health, they experience a higher level of fear and, thus, a further motivation of showing protective behaviors. Sharing facts about skin cancer and perceiving the real threat of skin cancer is essential for those at a frequent exposure to UVR of sunlight. Eliciting fear has a dramatic effect on changing attitude and behavior. Increasing fear up to a certain level increases the motivation for protection.

## 11. Strengths and Limitations of Research

One strength of the present research was the use of maximum variety of participants' age. Thus, generalization of results is to a great extent possible to the population of sailors. Observing their workplace and constant presence in fishing spots and seeing them using protectives against sunlight at work can be one way of confirming findings and describing the protective measures more in depth.

Sailors, as among external workers, have a lower level of education and literacy compared to other general populations. One limitation of the present research is that it only explored sailors' perceptions and experiences. Therefore, to achieve a more realistic and inclusive insight into the topic of research, it is suggested that future research is focused on dermatologists' experience or that of local coastal residents of the southern harbors of Iran. Another limitation of this research was a lack of cooperation of some sailors, which was tackled as far as possible by explaining the purpose of research and ensuring the confidentiality of the information they provided.

## 12. Conclusions

Sailors are at the risk of skin cancer due to their work conditions. In the present research, sailors' perceptions and determinants of skin cancer and protective behaviors against sunlight were identified. Promoting protective behaviors and changing beliefs that hindering preventive behaviors are among issues that need special attention. Thus, preventive strategies should focus on corrected beliefs and promoted protective behaviors for primary preventive measures. Social interactions should be strengthened through encouraging and educating sailors routinely by healthcare providers including doctors as an effective strategy. Regular screening and examination should be done for sailors and integrated into their health programs. Screening and increasing self-care can reduce sailors' exposure to UVR in sunlight and, thus, lower the chances of affliction with skin cancer in this vulnerable population.

## Figures and Tables

**Table 1:** Sailors' demographic information.

Variable	*n*. (%)
Age	
20–30 years	7 (30)
31–40 years	10 (43)
41–50 years	6 (26)

Education	
Below diploma	14 (60)
Diploma	7 (30)
University	2 (9)

Marital status	
Married	17 (73)
Single	4 (17)
Divorced	2 (9)

Work experience	
Below 5 years	6 (26)
5–10 years	10 (43)
10 years or more	7 (30)

Insurance	
Yes	16 (69)
No	7 (30)

History of sunburn	
Yes	9 (39)
No	14 (61)

**Table 2:** Codes, categories, and subcategories of sailors' perceptions and experiences of skin cancer and protective behaviors.

Category	Subcategory	Code
Protective behaviors	Change of work time	Activities at night
		Fewer activities at the peak time of sunlight
		Activities in the evenings
	Use of protectives	Wearing hats while working
		Wearing sunscreen
		Wearing long sleeves

Hard personal and familial conditions	Loss of peace	Feeling depressed
		Disappointment and loneliness
		Misgivings
		Weakness
	Loss of work and family status	Loss of status in family
		Problem earning a living
	Loss of health	Incurability of cancer
		Injuries caused by cancer
		Cancer and death

Social interactions	Demotivation and discouragement	Unwillingness to show protective behaviors
		Doctors' prohibition
		Ineffectiveness of protective behaviors in preventing skin cancer
	Rejection	Friends' criticism
		Being humiliated

Poor social support	Issues with living	High cost of sunscreen
		Priority of earning a living
		Family responsibility
	Negative attitude to protective behaviors	Wearing sunscreen damages skin.
		Wearing sunscreen hurts eyes.
		Wearing long sleeves in hot weather leads to red skin or blisters.

Feeling of satisfaction	Feeling healthy by protective behaviors	Feeling healthy while wearing long sleeves
		Feeling healthy with less sun-burned head and neck while wearing veiled hats
		Feeling healthy when less exposed to sunlight at the peak time
	Preventing the development of disease and lowering costs	Saving money and lowering medical costs by timely visits
		Lowering costs of sunburns while wearing hats
		Hope to be cured by timely visits

Self-care	Capability of self-care	Wearing long sleeves most of the day
		Wearing hats and scarves during intense sunshine
		Reducing the exposure time to sunlight in most days
	Strengthening beliefs	Attempts to raise awareness and knowledge of preventing skin cancer
		Attempts to gain information in doctor-patient visits in future

Fear	Fear of affliction	Fear of positive results
		Concerns about skin examination and diagnosis of cancer
		Fear of talking about skin cancer
	Continuous concerns	Disrupted ordinary life due to cancer
		Loneliness and isolation due to cancer
		Isolation from family and friends
		Fear of affliction with skin cancer due to recurrent exposure to sunlight

## Data Availability

The data used to support the findings of this study are available from the corresponding author upon request. Thus, this study is a qualitative study.
